# Thermoelectric properties of sorted semiconducting single-walled carbon nanotube sheets

**DOI:** 10.1080/14686996.2019.1567107

**Published:** 2019-03-01

**Authors:** Wenxin Huang, Eriko Tokunaga, Yuki Nakashima, Tsuyohiko Fujigaya

**Affiliations:** a Department of Applied Chemistry, Graduate School of Engineering, Kyushu University, Fukuoka, Japan; b The World Premier International Research Center Initiative, International Institute for Carbon Neutral Energy Research (WPI-I2CNER), Kyushu University, Fukuoka, Japan; c Japan Science and Technology Agency (JST-PRESTO), Kawaguchi, Japan; d Center for Molecular Systems (CMS), Kyushu University, Fukuoka, Japan

**Keywords:** Semiconducting carbon nanotubes, thermoelectric conversion, figure of merit, thermal conductivity, Seebeck coefficient, 50 Energy Materials, 104 Carbon and related materials, 105 Low-Dimension (1D/2D) materials, 206 Energy conversion / transport / storage / recovery, 210 Thermoelectronics / Thermal transport / insulators

## Abstract

Single-walled carbon nanotubes (SWNTs), especially their semiconducting type, are promising thermoelectric (TE) materials due to their high Seebeck coefficient. In this study, the in-plane Seebeck coefficient (*S*), electrical conductivity (*σ*), and thermal conductivity (*κ*) of sorted semiconducting SWNT (s-SWNT) free-standing sheets with different s-SWNT purities are measured to determine the figure of merit *ZT.* We find that the *ZT* value of the sheets increases with increasing s-SWNT purity, mainly due to an increase in Seebeck coefficient while the thermal conductivity remaining constant, which experimentally proved the superiority of the high purity s-SWNT as TE materials for the first time. In addition, from the comparison between sorted and unsorted SWNT sheets, it is recognized that the difference of *ZT* between unsorted SWNT and high-purity s-SWNT sheet is not remarkable, which suggests the control of carrier density is necessary to further clarify the superiority of SWNT sorting for TE applications.

## GRAPHICAL ABSTRACT

**Figure:**
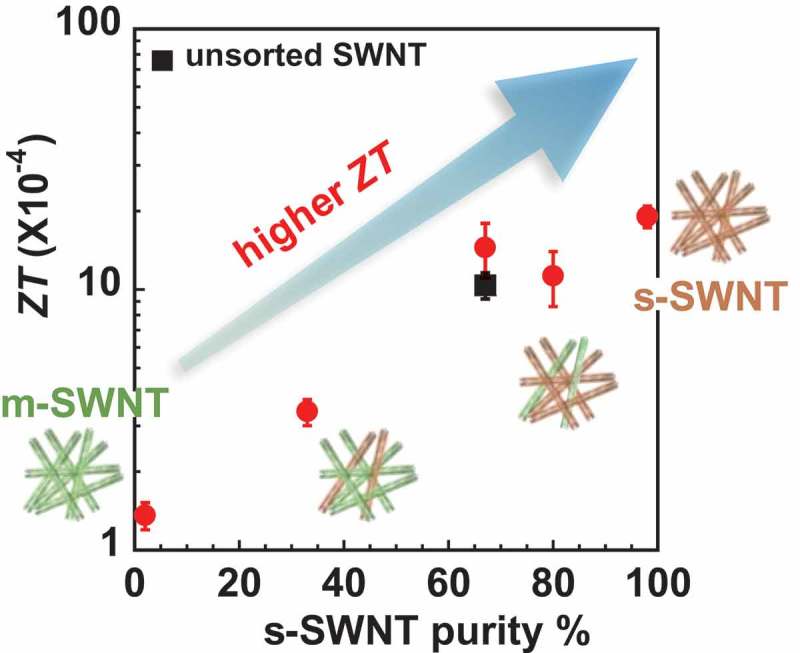

## 1. Introduction

As wearable sensors gain in demand, flexible, compact, lightweight, low-cost power supplies that can be used without charging are in great need [1]. In such a wearable battery, thermoelectric (TE) conversion is convenient because it is simple in principle and less limited in energy source [2]. The efficiency of TE conversion is defined by the figure of merit (*ZT*), which is calculated as *ZT = (σS^2^/κ)T*, where *S, σ, κ*, and *T* are the Seebeck coefficient (V K^−1^), electrical conductivity (S m^−1^), thermal conductivity (W m^−1^ K^−1^), and absolute temperature (K), respectively [3]. Thus, improving TE conversion efficiency requires materials with higher Seebeck coefficient and electrical conductivity as well as lower thermal conductivity. Although conventional TE materials such as bismuth, tellurium, and antimony have high *ZT* values over 1.0 at room temperature [4,5], these materials are relatively toxic, rare, and difficult to process [6], which hinders their applications in wearable and flexible TE devices.

Recently, various conductive polymers [7] such as poly(3,4-ethylenedioxythiophene) doped with polystyrene sulfonate (PEDOT/PSS) [8], and acid-doped polyaniline [9–11], together with carbon nanotubes (CNTs) [12] have attracted attention as TE materials due to their electrical conductivity, lightness, flexibility, low toxicity, abundance, and production scalability [13]. In particular, semiconducting single-walled carbon nanotubes (s-SWNTs) have attracted strong attention as a promising TE material because of their large Seebeck coefficient over 1000 μV K^−1^ at room temperature [14–17], which is much higher than inorganic semiconducting materials [18]. In reality, most SWNTs are produced as a 1:2 mixture of metallic (m-) and s-SWNTs [19,20] and need to be extracted or sorted to use s-SWNTs. Recently, various methods such as gel chromatography [21], polyfluorene (PFO)-based polymer wrapping [22,23], DNA recognition [24], density gradient ultracentrifugation (DGU) [25,26] and two-phase separation [27,28] allow us to obtain s-SWNTs and to study their TE properties including their Seebeck coefficient, electrical conductivity, and thermal conductivity. Nakai et al. prepared s-SWNT sheet (thickness; 50–130 μm) using DGU technique and found that the s-SWNT sheet with 100% s-SWNT purity^1^ had a large Seebeck coefficient of 170 μV K^−1^, which was much higher than that of m-SWNT sheet (<25 μV K^−1^) and comparable to that of inorganic semiconducting materials [29]. They revealed that the Seebeck coefficient was increased as the purity of s-SWNT increased [29], which was also systematically studied by Piao et al. [30]. For the s-SWNT sheets, it was demonstrated that Seebeck coefficient was further increased by optimizing the doping level chemically [14,16,31,32] or electrochemically [33–35] and, quite importantly, the higher purity of s-SWNT leads to a larger increase of Seebeck coefficient [29], thus the value reached to 2000 μV K^−1^ [14].

On the other hand, the electrical conductivity of s-SWNT was much lower than that of m-SWNT, and the electrical conductivity decreased as the s-SWNTs purity increased [36]. Such a negative correlation between Seebeck coefficient and electrical conductivity cannot be ruled out even under changing the carrier density; namely, the increase of electrical conductivity of the s-SWNT network from 10^3^ to 10^6^ S m^−1^ by chemical doping resulted in the large decrease of the Seebeck coefficient [14,31,32]. Thus, the power factor (*PF*) of the s-SWNT network, defined as *σS*^2^, has a maximum value when the electrical conductivity is around 10^4^–10^5^ S m^−1^ [14,31,32]. It has been revealed that the maximum *PF* can be improved by optimizing 1) the diameter of SWNT [14], 2) the morphology of the SWNT network including the bundle size, SWNT length [37–39], and 3) removal of dispersant [31,32]. Thus, the maximum *PF* of s-SWNT network can exceed 500 μW m^−1^ K^−2^ [31], which was much greater than that of m-SWNT network (<10 μW m^−1^ K^−2^) [29]. Although the dependences of Seebeck coefficient, electrical conductivity and *PF* on the s-SWNT purity and carrier density have been investigated as discussed above, *ZT* value has not been systematically studied, and the thermal conductivity was merely discussed for these samples. This lack of measurements might originate from difficulties in measuring thermal conductivity for thin SWNT network samples cast on a substrate. Besides, such measurements typically require a different setup than that used for evaluating Seebeck coefficient and electrical conductivity, and fall out of scope of many studies. Hence, reports on *ZT* in s-SWNTs are limited to selected samples [14,31,32] and present no systematic research as a function of s-SWNTs purity.

Here we investigated the *ZT* values of the s-SWNT sheets depending on the purity of s-SWNTs for the first time, where thermal conductivity was measured using free-standing s-SWNT sheets. In addition, to study the advantage of s-SWNT sheets for TE application, we also measured the TE properties of unsorted SWNT sheet to find out the effect of the sorting process.

## 2. Experimental section

### 2.1. Materials

s-SWNTs (98% IsoNanotubes-S), m-SWNTs (98%, IsoNanotubes-M), and raw SWNTs (PureTubes, arc discharge) were purchased from NanoIntegris. Sodium dodecyl benzenesulfonate (SDBS) was purchased from Tokyo Chemical Industry, and acetone and methanol were purchased from Kanto Chemical. All the chemicals were used as received. Milli-Q water with a resistivity higher than 18 MΩ cm was used.

### 2.2. Measurements

The in-plane electrical conductivity and in-plane Seebeck coefficient were measured using a ZEM-3 (ADVANCE RIKO, Japan) under a helium atmosphere at ~0.01 MPa from 30 to 100 °C. The specific heat capacity (*C_p_*) was measured by differential scanning calorimetry (DSC) using an EXSTAR DSC 6200 (SII Nanotechnology, Japan) at a heating rate of 10 K min^−1^. A certified sapphire crystal (Al_2_O_3_) was used as the reference sample. In-plane thermal diffusivities (*α*) were measured using a Thermowave Analyzer TA (Bethel, Japan). The density of the sheets (*ρ*) was calculated from their weight and volume. The thermal diffusivity was evaluated by periodic heating (a non-steady-state method) and thermal conductivity *κ* was calculated as *κ = C_p_∙α∙ρ*. Optical absorption and Raman spectroscopy were performed using a V-670 Spectrophotometer (JASCO) and a RAMAN RXN (Kaiser Optical Systems), respectively. A 785-nm laser was used as the excitation in Raman spectroscopy. Scanning electron microscopy (SEM) was carried out using SU-9000 (Hitachi High Technologies, 5.0 kV acceleration voltage). Atomic force microscopy (AFM) images were recorded using SPM-9600 system (Shimadzu Corporation).

### 2.3. Preparation of SWNT free-standing sheets

s-SWNTs and m-SWNTs were sonicated in an SDBS water solution (0.5%) using a bath sonicator (BRANSON, 5510) for 1 h and a probe sonicator (TOMY, UD-200) for 20 min. The total amount of s-SWNTs and m-SWNTs were controlled to be 3.0 mg, and the ratio of s-SWNTs and m-SWNTs depended on the final purity of the s-SWNT sheets. The dispersion was filtered through a mixed cellulose ester membrane (pore size of 0.2 μm, ADVANTEC), and the membrane was removed by dipping it in fresh acetone four times. The obtained sheet was dipped in water and methanol and then dried overnight. Unsorted SWNT sheet and s-SWNT sheets with s-SWNT purities of 98% and 2% were prepared using raw SWNTs, 98% s-SWNTs, and 98% m-SWNTs, respectively. s-SWNTs with purities of 80%, 67%, and 33% were prepared by mixing 98% s-SWNTs and 98% m-SWNTs in ratios of 4:1, 2:1, and 1:2, respectively. The thickness of the sheets was 20–30 μm.

## 3. Results and discussion

### 3.1. Preparation of s-SWNT sheets

**Figure 1.**
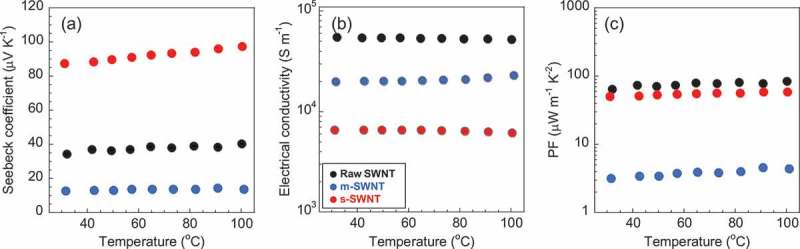
In-plane (a) Seebeck coefficient, (b) electrical conductivity, and (c) *PF* of the as-purchased raw SWNT sheet (black), s-SWNT sheet (red), and m-SWNT sheet (blue) from 30 to 100 °C in a helium atmosphere at 0.01 MPa.

Figure 1 shows the temperature dependences of the Seebeck coefficient (Figure 1(a)), electrical conductivity (Figure 1(b)), and *PF* (Figure 1(c)) of the raw SWNT, m-SWNT, and s-SWNT sheets. These samples showed no obvious temperature dependence in this temperature range. The s-SWNT sheet showed higher Seebeck coefficient than the raw SWNT sheet due to the higher s-SWNT purity than the raw SWNT sheet, while the m-SWNT sheet showed a lower Seebeck coefficient due to its lower s-SWNT purity compared with raw SWNT sheet, which agrees well with the reported results [29]. In the Raman spectra (Supporting information, Figure S1), a large decrease of Breit-Wigner–Fano (BWF) peak (near 1560 cm^−1^) originated from m-SWNT was observed for s-SWNT sheet, which proved the validity of our samples [40,41].

On the other hand, the m-SWNT sheet showed lower electrical conductivity than the raw SWNT sheet (Figure 1(b)). We assumed that an introduction of defects and shortening of the SWNTs during DGU sorting caused the lowering of the conductivity as pointed out previously [42], which is also supported by our Raman spectra (Supporting information, Figure S1) and AFM images (Supporting information, Figure S2). Such a lowering of the electrical conductivity is supported by the theoretical simulation as well [43].

**Figure 2.**
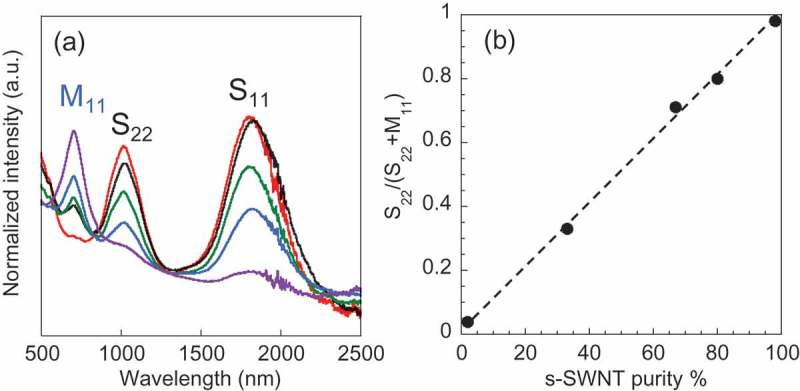
(a) UV-vis-NIR absorption spectra of s-SWNT dispersions with various s-SWNT purities: 98% (red), 80% (black), 67% (green), 33% (blue), and 2% (purple). (b) The S_22_ absorption peak area as a proportion of total peak area (S_22_+M_11_) as a function of the s-SWNT purity. The dotted lines was added as eye guide.

To study the TE properties of the sorted s-SWNT sheets without considering the effects of DGU sorting, we prepared s-SWNT sheets with 2%, 33%, 67%, 80% and 98% s-SWNT purities by mixing the sorted s-SWNTs and m-SWNTs in various ratios. Figure 2(a) shows the UV-vis-NIR absorption spectra of the SWNT dispersions with various s-SWNT purities. The broad absorption peaks centred at around 1800 nm (s-SWNT, S_11_ band) and 1000 nm (s-SWNT, S_22_ band) decreased in intensity as the s-SWNT purity decreased, while the peak at around 700 nm (m-SWNT, M_11_ band) increased in intensity. It is known that S_11_ peak is more sensitive to the doping level than S_22_ peak [44], therefore, the linear decrease of S_11_ intensity as the increase of s-SWNT purity suggested that the doping level of s-SWNT in these samples are almost identical. The ratio between S_22_ and M_11_ was utilized to estimate the purity of the s-SWNTs [30], where the linear relationship between S_22_/(S_22_+M_11_) and s-SWNT purity as plotted in Figure 2(b) indicates a good control of s-SWNT purity and guarantees the validity of the estimated purity (Supporting information, Table S1). From the SEM observations, the sheets were formed by SWNT bundles with a diameter around 20 nm of the SWNTs (Supporting information, Figure S3). SWNTs are bundled and entangled in the sheets, and hence their length is hard to measure precisely. From the AFM images, we evaluated the average length of isolated s-SWNTs, m-SWNTs and raw SWNTs as 1.1 ± 0.4, 0.9 ± 0.3 and 1.3 ± 0.6 μm (Figure S2). The density of the s-SWNT sheets with 2%, 33%, 67%, 80% and 98% purity was determined to be 0.46, 0.56, 0.64, 0.56, and 0.55 g cm^−3^, respectively, similar to the other report [16].

### 3.2. TE properties of s-SWNT sheets

**Figure 3.**
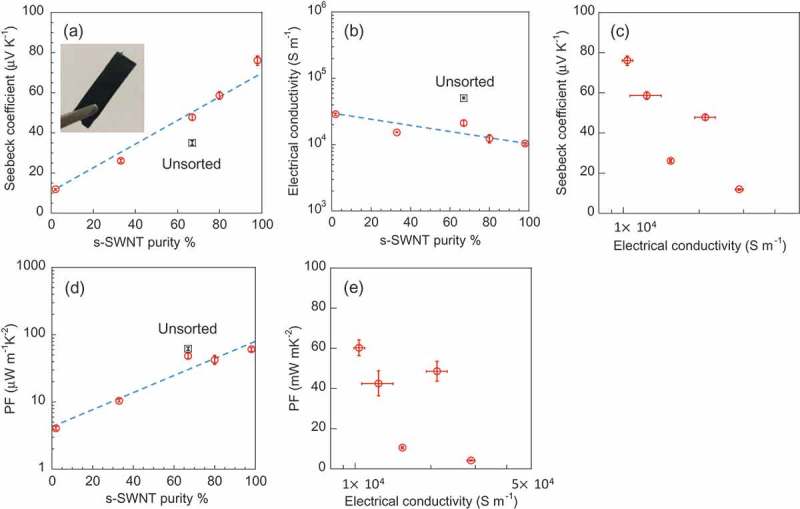
In-plane (a) Seebeck coefficient and (b) electrical conductivity as a function of the s-SWNT purity (red circles). (c) Seebeck coefficient as a function of electrical conductivity. *PF* of s-SWNT sheets as a function of (d) s-SWNT purity and (e) electrical conductivity. The TE values of the unsorted SWNT sheet are also plotted (black squares). Inset in (a) shows the photograph of free-standing 98% s-SWNT sheet. The blue dotted lines were added as eye guide.

Figure 3 summarizes the Seebeck coefficient, electrical conductivity, and *PF* of s-SWNT sheets at 30 °C as a function of s-SWNT purity. The unsorted SWNT sheet was also prepared using the raw SWNT in the same way to discuss the effect of the sorting process for TE properties. Inset in Figure 3(a) is the photograph of 98% s-SWNT sheet, which shows that all the samples used for measurements are free-standing sheets. As shown in Figure 3(a), the Seebeck coefficient of the s-SWNT sheets linearly increased with the increasing s-SWNT purity, which showed the same trend as previously reported [30], and reached 76.0 μV K^−1^ at 98% s-SWNTs. The trend was close to the serial model [45,46] given by *S* ≈ α^2^*S*_ss_ + (1 – α^2^)*S*_mm_, where *S*_ss_, *S*_mm_ and α represents Seebeck coefficient of s-SWNT/s-SWNT junction, Seebeck coefficient of m-SWNT/m-SWNT junction and fraction of s-SWNT (α: 0 ~ 1), respectively [29] (76.0 and 11.9 μV K^−1^ was used as *S*_ss_ and *S*_mm_, respectively). Lower Seebeck coefficient of the 98% s-SWNT sheets (76.0 μV K^−1^) compared to the reported value (~150 μV K^−1^) [29] for the comparable s-SWNT purity may come from the difference of the other parameters such as SWNT diameter [14,17], the bundle size of SWNTs [31,32,47], and the doping level [16,32,35]. The previously reported Seebeck coefficient values of s-SWNT sheets are summarized in Table S2 as a reference.

Meanwhile, as shown in Figure 3(b), the electrical conductivity of the sheets decreased as the s-SWNT purity increased, reaching 1.04 × 10^4^ S m^−1^ for the 98% s-SWNT sheet. On the other hand, the Seebeck coefficient and electrical conductivity show a negative correlation (Figure 3(c)), which is the same as previous report [14,31,32]. As a result, the *PF* (=*σS*^2^) increased as the s-SWNT purity increased (Figure 3(d)). Interestingly, we recognized that the *PF* values were decreased with the increasing electrical conductivity as plotted in Figure 3(e). The trend is opposite from the trend for *PF* changes upon carrier doping as firstly reported by Nakai et al. [29]; namely, an increase of the electrical conductivity led to the increase of *PF* at low electrical conductivity region (<10^4^ S m^−1^) [14,29,31,32]. Although the reason was not discussed, Nakai et al. also reported the similar trend with our result; namely, the increase of *PF* as the decrease of the electrical conductivity in the comparison between m-, s-SWNT and their mixture [29]. Above results suggested that *S*^2^ is dominant for *PF* when the s-SWNT purity is controlled, whereas *σ* is dominant when carrier density is controlled.

Notably, we recognized the *PF* of the unsorted SWNT sheet (61.6 μW m^−1^ K^−2^) was almost comparable to that of the 98% s-SWNT sheet (60.3 μW m^−1^ K^−2^) as plotted in Figure 3(d). This result occurred because the unsorted SWNTs had much higher electrical conductivity than s-SWNT sheets (Figure 3(b)) since unsorted SWNTs was less damaged than sorted SWNTs as discussed above [42,48–50]. Indeed, in the Raman spectra of the s-SWNT sheet, the D-band intensity at 1307.4 cm^−1^ normalized by the G-band intensity at 1592.4 cm^−1^ was higher than that of the unsorted SWNT sheet (Supporting Information, Figure S4) [51].

**Figure 4.**
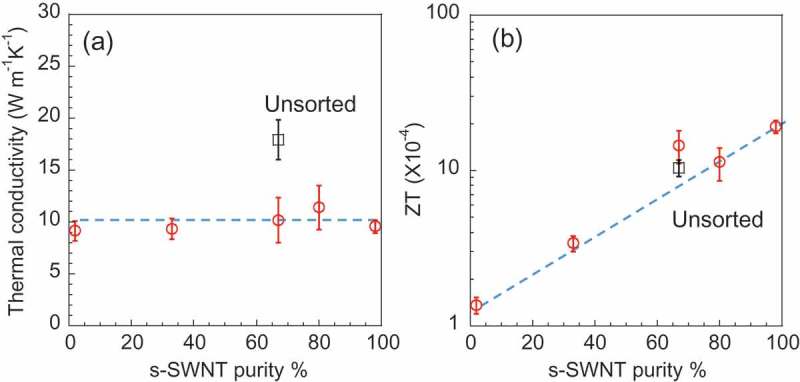
In-plane (a) thermal conductivity and (b) *ZT* of s-SWNT sheets as a function of the s-SWNT purity (red circles). The TE values of the unsorted SWNT sheet are also plotted (black squares). The blue dotted lines were added as eye guide.

Figure 4(a) shows thermal conductivity of the sheets as functions of s-SWNT purity (for thermal diffusivity, see Supporting Information, Figure S5). We found that the thermal conductivity of the s-SWNT sheets remained constant as the s-SWNT purity changed, which is in accordance with the theoretical calculation of a single m- and s-SWNT [52] and experimental result [42] of m- and s-SWNT thin films. In thermal conduction of SWNTs, it was proved that the phonon transport mainly contributed to the thermal conductivity and electron transport could be ignored in room temperature [52]. The in-plane thermal conductivity of SWNT sheets varied within two orders of magnitude in the literatures as summarized in Table S3. Therefore, the difference of the thermal conductivity range between our results (9.16–17.9 W m^−1^ K^−1^) and reported values (80–370 W m^−1^ K^−1^) was probably due to the difference of either SWNT diameter [53], length [54,55], bundle size [56], anisotropy [57,58], defect density [43], mass density [59] of SWNTs or measurement accuracy [42,60–62]. We also recognized that the s-SWNT sheets had lower thermal conductivity than that of the unsorted SWNT sheet. Similar to the electrical conductivity, it can also be explained by the introduction of defects (Supporting Information, Figure S4) or the shortening of SWNTs upon sorting, which leads to additional phonon scattering in the defective regions of SWNTs and the SWNT junctions inside the sheets as predicted by theoretical simulations [43,63,64].

### 3.3. ZT values of s-SWNT sheets

Based on the above measurements, the in-plane *ZT* values of the s-SWNT sheets were calculated (Figure 4(b)). We found that *ZT* value was increased with the increasing s-SWNT purity and reached 1.91 × 10^−3^ at 98% s-SWNT. This value is 14 times higher than the *ZT* at 2% s-SWNT (1.36 × 10^−4^), which clearly demonstrates that higher purity of s-SWNTs is beneficial for TE materials. In addition, *ZT* of the 98% s-SWNT sheet was higher than that of the unsorted SWNTs (1.04 × 10^−3^), supporting the significance of s-SWNT sorting for TE applications. However, it is also important to recognize that difference between 98% s-SWNT and unsorted SWNT sheets in *ZT* was not remarkable similarly to the difference of *PF* between 98% s-SWNT sheet and unsorted SWNT sheet. On the other hand, Hayashi et al. reported the sorted s-SWNT provided higher Seebeck coefficient compared with the unsorted SWNT when the carrier doping was controlled [16], which demonstrated the superiority of s-SWNTs over the unsorted SWNTs. Therefore, to further verify the superiority of s-SWNT for TE application, it is necessary to systematically study the *ZT* value of high purity s-SWNT sheets depending on their carrier doping level [14,16,31,32]. It is known that the carrier doping can control the Fermi level of s-SWNT sheets [14,31,32]. Since Seebeck coefficient depends on the Fermi level position, maximizing of the Seebeck coefficient of the SWNT sheets is possible by carrier doping [14,31,32], which provides a larger *ZT* value. Previously, Ferguson et al. systematically studied *PF* values and achieved ~700 μW m^−1^ K^−2^ for high-purity s-SWNT networks by chemical carrier doping [14,31,32]. With the proper characterizations of thermal conductivity, it is possible to systematically study the *ZT* for high purity s-SWNTs to determine optimal *ZT*.

## 4. Conclusions

We measured the TE properties of s-SWNT free-standing sheets with various s-SWNT purities to systematically evaluate their *ZT* values. Measurements of the Seebeck coefficient, electrical conductivity and thermal conductivity with the same samples enabled such a systematic evaluation. We confirmed the previous theoretical prediction that the thermal conductivity of s-SWNT sheets does not depend on the s-SWNT purity. As a result, we found that *ZT* value increased with the increasing s-SWNT purity mainly due to the increase in the Seebeck coefficient, which is important for TE applications of SWNTs. Since the higher purity of s-SWNT leads to a larger increase of Seebeck coefficient upon controlling of carrier density, systematic studies of *ZT* value of s-SWNT sheets as a function of the carrier doping level are necessary. In addition, experimental investigations on the TE properties between sorted and unsorted SWNTs are also useful to determine the effects of defects induced by sorting process.

## Note

1. Purity means the content of s-SWNTs in SWNTs. 100% s-SWNT purity indicates that there is no m-SWNTs.
