# Supplementary material for: Thermoelectric properties of sorted semiconducting single-walled carbon nanotube sheets
Source: Sci Technol Adv Mater. 2019 Mar 1;20(1):97–104. doi: 10.1080/14686996.2019.1567107 (PMC6454402; doi:10.1080/14686996.2019.1567107)
Supplement: Supplemental Material [file TSTA_A_1567107_SM6766.zip › suppl_data/STAM (Huang) supporting 20181208 revised.pdf]

Supporting Information for

Thermoelectric properties of sorted semiconducting single-walled carbon nanotube sheets

Wenxin Huang[a], Eriko Tokunaga[a], Yuki Nakashima[a] and Tsuyohiko Fujigaya[a,b,c,d]*

[a]Department of Applied Chemistry, Graduate School of Engineering, Kyushu University, 744 Motooka, Nishi-ku, Fukuoka 819-0395, Japan; [b]The World Premier International Research Center Initiative, International Institute for Carbon Neutral Energy Research (WPI-I2CNER), Kyushu University, 744 Motooka, Nishi-ku, Fukuoka 819-0395, Japan; [c]JST-PRESTO, 4-1-8 Honcho, Kawaguchi, Saitama 332-0012, Japan; [d] Center for Molecular Systems (CMS), Kyushu University, 744 Motooka, Nishi-ku, Fukuoka 819-0395, Japan

**E-mail: fujigaya.tsuyohiko.948@m.kyushu-u.ac.jp*

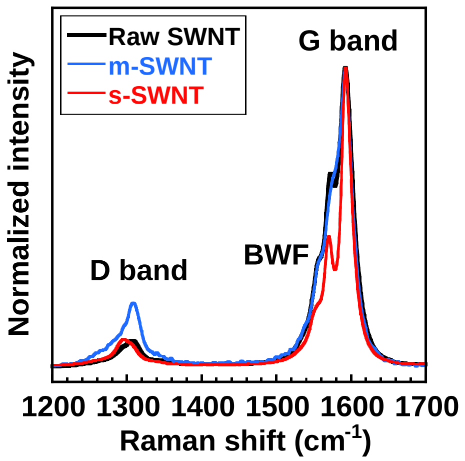

Figure S1. Raman spectra of as-purchased raw SWNTs (black), m-SWNTs (blue), and s-SWNTs (red) at an excitation wavelength of 785 nm.

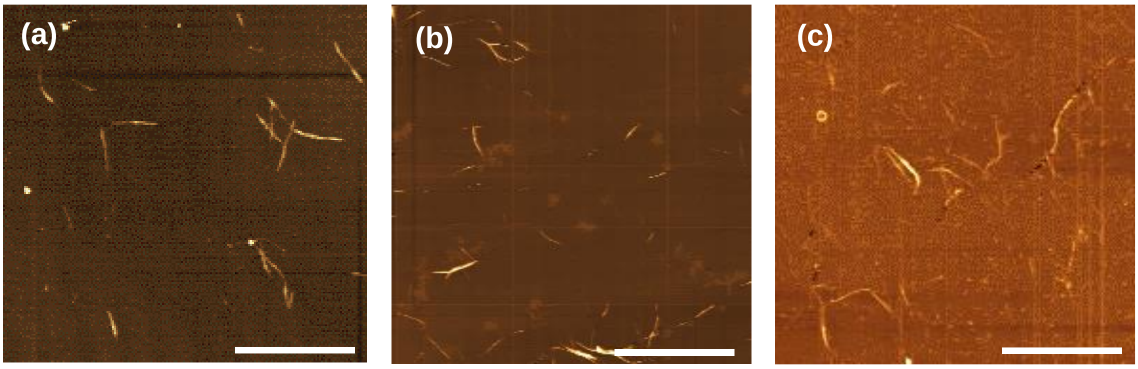

Figure S2. AFM images of as-purchased (a) s-SWNTs, (b) m-SWNTs, and (c) raw SWNTs with an average length of 1.1 ± 0.4, 0.9 ± 0.3 and 1.3 ± 0.6 μm, respectively. Scale bars; 5 μm.

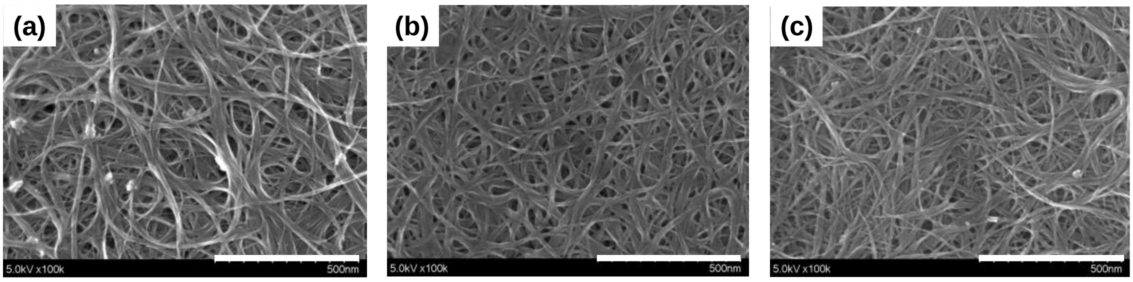

Figure S3. SEM images of s-SWNT sheets with s-SWNT purity of (a) 98%, (b) 67%, and (c) 2%. Scale bars; 500 nm.

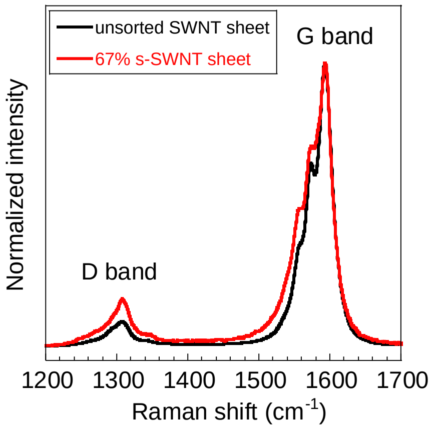

Figure S4. Raman spectra of unsorted SWNT (black) and 67% s-SWNT sheets (red) at an excitation wavelength of 785 nm.

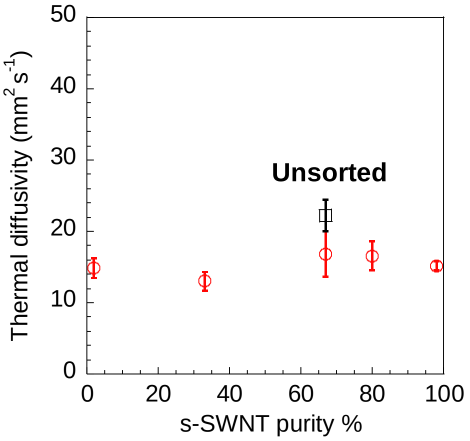

Figure S5. In-plane thermal diffusivity of s-SWNT sheets as a function of the s-SWNT purity (red circles) and the unsorted SWNT sheet (black square).

Table S1. S_{22} and M_{11} peak areas, calculated from UV-vis-NIR absorption in Figure 2 after peak fitting

	98% s-SWNT	80% s-SWNT	67% s-SWNT	33% s-SWNT	2% s-SWNT
M_{11} area	0.75	8.36	9.94	21.27	21.60
S_{22} area	39.88	33.51	23.87	10.31	0.80
S_{22}/M_{11}	52.88	4.01	2.40	0.48	0.04
$S_{22}/(S_{22}+M_{11})$	0.98	0.80	0.71	0.33	0.04
Fitting Type	Gauss	Gauss	Gauss	Gauss	Gauss

Table S2. In-plane Seebeck coefficients of p-type[a)] s-SWNT sheets at 30 °C in the literatures.

Type of SWNT	Method of extraction	Diameter (nm)	s-SWNT purity	Bundle size (nm)	Dopant	S (μV K^{-1})	Ref.
Calculated	–	0.5	100%	–	–	>2000	[S1]
	–	1.4	100%	–	–	800	[S1]
	–	0.8	100%	–	–	1285	[S2]
	–	1.3	100%	–	–	705	[S2]
Arc Discharge	DGU	1.4 (±0.2)	98%	19.6 (±11.3)	O_2	76.0	This study
	DGU	1.4	>99%	–	O_2	88	[S3]
	DGU	1.44	100%	–	O_2 dedoped	170	[S4]
	DGU	1.44	98%	–	O_2 dedoped	150	[S4]
	DGU	1.44	>99%	–	Nitric acid	30–180	[S5]
Laser vaporization	PFO-based[b)]	1.3	>99%	–	OA[g)]	64–700	[S2]
	PFO-based[c)]	1.3	>99%	23.9 (±6.7)	OA[g)]	20–200	[S6]
	PFO-based[c)]	1.3	>99%	42.6 (±12.0)	OA[g)]	23–130	[S6]
Plasma-torch	PFO-based[d)]	1.0	>99%	15 (±5)	OA[g)]	20–200	[S7]
	PFO-baesd[e)]	1.0	>99%	20 (±4)	OA[g)]	21–90	[S7]

[a)]refers to holes as the main transport carrier in the s-SWNT. [b)]poly[(9,9-dioctylfluorenyl-2,7-diyl)-*altco*-(6,6′-(2,2′-bipyridine)]; [c)]1,1′-(((1E,1′E)-(9,9-didodecyl-9H-fluorene-2,7-diyl)bis(ethene-2,1-diyl))bis(6-methyl-4-oxo-1,4-dihydropyrimidine-5,2-diyl))bis(3-dodecylurea); [d)]poly[(9,9-di-n-dodecyl-2,7-fluorendiyl-dimethine)-(1,4-phenylene-dinitrilomethine)]; [e)]1,10-(((1E,10E)-(9,9-didodecyl-9H-fluorene-2,7-diyl)bis(ethene-2,1-diyl))bis(6-methyl-4-oxo-1,4-dihydropyrimidine-5,2-diyl))bis(3-dodecylurea); [f)]double-walled carbon nanotube; [g)]triethyloxonium hexachloroantimonate; [h)]chlorosulfonic acid;

Table S3. In-plane thermal conductivities of SWNT sheets at 30 °C in the literature

Orientation of SWNT networks	Length of SWNTs (μm)	Density (g cm^{-3})	Measurement techniques			κ (W m^{-1} K^{-1})	Ref.
			Steady-state or non-steady-state method	Heating method	Detection method		
Random	0.6–1.9	0.46–0.76	Non-steady	Periodic heating	Phase difference of temperature wave	9.16–17.9	This study
	0.5–1.0	0.5–1.1	Steady	Electrical heating	IR thermal imaging (temperature)	80–370	[S8]
	1.0	–	Steady	Laser Beam (Raman spectrometer)	Raman spectra	26	[S9]
	–	0.90, 1.35	Non-steady	Periodic heating	Phase difference of temperature wave	9.8 (±3.3), 39 (±12)	[S10]
	–	–	Steady	Laser Beam (Raman spectrometer)	Raman spectra	18.3	[S11]
	5.2 (±0.7)	1.5 (±0.2)	Steady	IR radiation by light emitting diode (Bolometric technique)	Si diode temperature sensor	75	[S12]
	1–10	0.509	Non-steady	Periodic heating	Phase difference of temperature wave	24.4	[S13]
	–	–	Steady	Not indicated	Comparative method (with constantan)	15, 17.5	[S14]
	–	0.42	Steady	Electrical heating	Record temperature as a function of applied power	2.2	[S2]
	–	–	Steady	Electrical heating	Record temperature as a function of applied power	1.39 (±0.43), 2.38 (±0.98)	[S7]
	–	–	Steady	Electrical heating	Record temperature as a function of applied power	2.45–3.85	[S6]
	–	–	Steady	Electrical heating (Self-heating method)	Calculation from current and resistance plot	18, 24	[S15]
	–	1.1	Steady	Electrical heating (Self-heating method)	Calculation from current and resistance plot	43 (±4)– 51 (±5)	[S16]
	>1.0	–	Steady	Not indicated	Comparative method (with constantan)	2.3, 35	[S17]
	–	–	Steady	Heat flow by PPMS a)	Temperature difference (by Thermometer)	2.6	[S18]
	–	1.33	Steady	Not indicated	Comparative method (with constantan)	30	[S19]
Oriented	–	1.33	Steady	Not indicated	Comparative method (with constantan)	220	[S19]
	–	–	Steady	Not indicated	Comparative method (with constantan)	42	[S20]
	–	0.6–0.9	Steady	Not indicated	Comparative method (with constantan)	60	[S21]

a) physical property measurement system

Reference

[S1] Hung NT, Nugraha ART, Hasdeo EH, et al. Diameter dependence of thermoelectric power of semiconducting carbon nanotubes. Phys Rev B. 2015;92:165426.

[S2] Avery AD, Zhou BH, Lee J, et al. Tailored semiconducting carbon nanotube networks with enhanced thermoelectric properties. Nature Energy. 2016;1:16033.

[S3] Piao M, Joo M-K, Na J, et al. Effect of Intertube Junctions on the Thermoelectric Power of Monodispersed Single Walled Carbon Nanotube Networks. J Phys Chem C. 2014;118:26454-26461.

[S4] Nakai Y, Honda K, Yanagi K, et al. Giant Seebeck coefficient in semiconducting single-wall carbon nanotube film. Appl Phys Express. 2014;7:025103.

[S5] Hayashi D, Ueda T, Nakai Y, et al. Thermoelectric properties of single-wall carbon nanotube films: Effects of diameter and wet environment. Appl Phys Express. 2016;9:025102.

[S6] Norton-Baker B, Ihly R, Gould IE, et al. Polymer-Free Carbon Nanotube Thermoelectrics with Improved Charge Carrier Transport and Power Factor. ACS Energy Letters. 2016;1:1212-1220.

[S7] MacLeod BA, Stanton NJ, Gould IE, et al. Large n-and p-type thermoelectric power factors from doped semiconducting single-walled carbon nanotube thin films. Energy Environ Sci. 2017;10:2168-2179.

[S8] Lian F, Llinas JP, Li Z, et al. Thermal conductivity of chirality-sorted carbon nanotube networks. Appl Phys Lett. 2016;108:103101.

[S9] Duzynska A, Taube A, Korona KP, et al. Temperature-dependent thermal properties of single-walled carbon nanotube thin films. Appl Phys Lett. 2015;106:183108.

[S10] Nonoguchi Y, Nakano M, Murayama T, et al. Simple Salt-Coordinated n-Type Nanocarbon Materials Stable in Air. Adv Funct Mater. 2016;26:3021-3028.

[S11] Sahoo S, Chitturi VR, Agarwal R, et al. Thermal Conductivity of Freestanding Single Wall Carbon Nanotube Sheet by Raman Spectroscopy. ACS Appl Mater Interfaces. 2014;6:19958-19965.

[S12] Itkis ME, Borondics F, Yu A, et al. Thermal Conductivity Measurements of Semitransparent Single-Walled Carbon Nanotube Films by a Bolometric Technique. Nano Lett. 2007;7:900-904.

[S13] Nakashima Y, Nakashima N, Fujigaya T. Development of air-stable n-type single-walled carbon nanotubes by doping with 2-(2-methoxyphenyl)-1,3-dimethyl-2,3-dihydro-1H-benzo[d]imidazole and their thermoelectric properties. Synth Met. 2017;225:76-80.

[S14] Vavro J, Llaguno MC, Satishkumar BC, et al. Electrical and thermal properties of C 60-filled single-wall carbon nanotubes. Appl Phys Lett. 2002;80:1450-1452.

[S15] Zhou W, Fan Q, Zhang Q, et al. High-performance and compact-designed flexible thermoelectric modules enabled by a reticulate carbon nanotube architecture. Nat Commun. 2017;8:14886.

[S16] Zhou W, Fan Q, Zhang Q, et al. Ultrahigh-Power-Factor Carbon Nanotubes and an Ingenious Strategy for Thermoelectric Performance Evaluation. Small. 2016;12:3407-3414.

[S17] Hone J, Whitney M, Piskoti C, et al. Thermal conductivity of single-walled carbon nanotubes. Phys Rev B. 1999;59:R2514-R2516.
[S18] Nonoguchi Y, Hata K, Kawai T. Dispersion of Synthetic MoS2 Flakes and Their Spontaneous Adsorption on Single-Walled Carbon Nanotubes. ChemPlusChem. 2015;80:1158-1163.
[S19] Hone J, Llaguno MC, Nemes NM, et al. Electrical and thermal transport properties of magnetically aligned single wall carbon nanotube films. Appl Phys Lett. 2000;77:666-668.
[S20] Gonnet P, Liang Z, Choi ES, et al. Thermal conductivity of magnetically aligned carbon nanotube buckypapers and nanocomposites. Current Applied Physics. 2006;6:119-122.
[S21] J. E. Fischer, W. Zhou, J. Vavro, et al. Magnetically aligned single wall carbon nanotube films: Preferred orientation and anisotropic transport properties. J Appl Phys. 2003;93:2157-2163.
